# The molecular insights of cyanobacterial bioremediations of heavy metals: the current and the future challenges

**DOI:** 10.3389/fmicb.2024.1450992

**Published:** 2024-10-11

**Authors:** Jinita Lourembam, Banaraj Haobam, Kshetrimayum Birla Singh, Savita Verma, Jay Prakash Rajan

**Affiliations:** ^1^Department of Zoology, School of Life Sciences, Manipur University, Canchipur, India; ^2^Department of Biotechnology, Kamakhya Pemton College, Hiyangthang, -Imphal, India; ^3^Chemistry Department, School of Engineering, Presidency University, Bengaluru, India; ^4^Department of Chemistry, Pachhunga University College, Mizoram University, Aizawl, India

**Keywords:** water pollution, biosorption, sustainability, human health, toxicity

## Abstract

In recent years, overexplorations of ore and the growth of industries are the prime factors in the release of heavy metals in environments. As a result, the food crops and water bodies are contaminated with metals which may have several adverse effects on the health of humans and other living species. These metals and metalloids, such as Zn, Cu, Mn, Ni, Cr, Pb, Cd, and As, upset the biochemical pathways of metabolite synthesis in living organisms and contribute to the etiology of different diseases. Microorganisms include bacteria, archaea, viruses, and many unicellular eukaryotes, which can span three domains of life—Archaea, Bacteria, and Eukarya—and some microorganisms, such as cyanobacteria, have shown high efficiency in the biosorption rate of heavy metals. Cyanobacteria are suitable for bioremediation as they can grow in adverse environments, have a less negative impact on the surrounding environment, and are relatively cheaper to manage. The structure of cyanobacteria has shown no extensive internal-bound membranes, so it can directly employ the physiological mechanisms to uptake heavy metals from contamination sites. Such biochemical makeups are suitable for managing and bioremediating heavy metal concentrations in polluted environments. This review aims to explore the potential of cyanobacteria in the bioremediation of heavy metals and metalloids in water bodies. Additionally, we have identified the prospects for enhancing bioremediation effectiveness.

## Introduction

Although heavy metals occur naturally all over the lyre of the earth’s soil, contamination in the ecosystem is because of the activities, mainly anthropogenic activities such as mining and coal mining, and industries related to agriculture, paints, batteries, automobiles, etc. The heavy metals are unable to degrade or be lost, so they can be toxic—even at very low concentrations—harmful to living beings, and reduce soil fertility. Heavy metal pollution in the environment originated from intensive anthropogenic and industrial operations, such as mining, electroplating, and refining (Vareda et al., 2019; Kamarudzaman et al., 2015). Agricultural activity, paper making, printing, electronic industries, traffic activity, and daily life activities such as household-generated waste, natural weathering of sediments, and volcanic activities release heavy metals into the environment. Thus, these released metals, such as Zn, Mn, Fe, Cr, Cu, Pb, Cd, Hg, and As, reach the soil and water above the recommended limit of the environmental protection agencies (Karn, 2016; Liu et al., 2023). Various anthropogenic activities, such as the burning of coal, leather tanning, preservatives, pesticides and pigment industries, refining industries, dye industries, etc., become the sources of the contamination of heavy metals mentioned *per se* and pose a threat to the environment (Aziz et al., 2023; Chabukdhara and Nema, 2012; Zhou et al., 2022). As per the World Health Organization (WHO) norms, the recommended levels of heavy metal should range from 0.05 to 0.2 mg/kg in the various drinking and edible food items (Das et al., 2023; Karn, 2016).

Excessive anthropogenic activity also alters the composition of soil, air, and water as they substantially impact the biological, chemical, and physical components (Khan et al., 2019; Mandre, 2014). For instance, an overabundance and advancement of industrial development, which is thought to be a significant contributing factor to global warming, could alter the heavy metal leaching pattern. Glaciers will melt and release heavy metals into the ocean as the temperature rises. The metal is also deposited via gravity deposition and is discharged by exhaust fumes as particulate particles. As a result, heavy metals, such as cadmium, lead, and mercury, directly enter our food chain, and their bioavailability to living organisms gradually rises (Xiao et al., 2024).

Due to the creation of novel technologies, research on heavy metal removal from contaminated soil and wastewater has advanced significantly in recent years. Many chemical, physical, and biological methods for eliminating heavy metals are now widely used due to these developments. The utilization of synthetic metal–organic framework compounds in heavy metal remediation has increased recently. They possess a substantial porosity and contain host compounds that let in visiting species, including heavy metal ions, into the cavity of their bulk structure (Rakesh et al., 2021). The pore size and geometry of metal–organic framework compounds are also selective for specific guest molecules from wastewater (Ramu et al., 2024). Techniques such as chemical precipitation and coagulation are frequently employed in the commercial sector to remove heavy metals from membranes and sorbents. However, there are several drawbacks to these methods, including limited removal efficiency, regeneration, high energy costs, and challenging byproduct management for chemical sludge (Wang et al., 2023; Femina et al., 2023). Consequently, researchers are looking for reasonably priced biomaterials or microorganisms to sequester heavy metals from wastewater and soil.

In the last two decades, studies have shown that heavy metals bioremediation from soil and water bodies is best accomplished by suitable sorbents of biomaterials, which are sustainable, low cost, easily available, ready to use after minor modification and high removal efficiency. For example, the heavy metal removal efficiency of *Moringa oleifera* seeds is more than 50%, and the main active component in *Moringa* seeds is found as a catalytic polyelectrolyte, and it exhibits a strong affinity for suspended heavy metals (Gomes et al., 2022). Similarly, the bioactive component tannin found in plants, such as tea, oak, and grapes, is a naturally occurring substance that contains polyphenolic compounds, and it can bond with heavy metals in wastewater. This substance has a great affinity for heavy metals and functions as a natural coagulant. Amino groups in animal-based biopolymers, including chitosan, actively bind to heavy metals.

Proteins are organic substances made up of amino acids. The biological materials that are intermediates between amino acids and proteins are known as peptides with an amino group (NH_2_) and a carboxyl group (COOH) joined by a peptide bond. The functional groups of amino acids, such as the amino group, carboxyl group, and imino groups in the peptide chain, can react and remove them through chelation, reduction, and formation of complexes. Such peptides can also potentially eliminate metals from contaminated water. A recent study found that peptides have a unique role in the purification of tainted water. The heavy metal biosorption mechanisms are the different functional groups containing peptides, such as amino and carboxylic acids, which have specific binding sites and effectively bind to heavy metal ions. The peptide–metal complexes prevent and reduce the toxicity of heavy metals to cells. Additionally, these compounds precipitate and immobilize, reducing the hazardous metals’ mobility (Luo et al., 2024).

Microorganisms such as algae, bacteria, and fungi produce a variety of biomolecules, including extracellular polymeric compounds such as proteins, and polysaccharides. Heavy metals may be efficiently bound by these biomolecules, enabling their extraction from wastewater. Comparably, amine, hydroxyl, and carboxyl functional groups in an extracellular polymeric material show a strong affinity for metal cations in wastewater (Badawi et al., 2023). Within the realm of microorganisms, cyanobacterial species exhibit greater potential than other microorganisms, including bacteria and fungi. *Anabaena subcylindrica* and *Nostoc* sp. are two examples of cyanobacteria that have previously demonstrated their promise as a removal agent for harmful metals from wastewater, including lead (Pb), cobalt (Co), manganese (Mn), and iron (Fe) (Ghorbani et al., 2022; Kalita and Baruah, 2023). Therefore, cyanobacteria have been at the center of research for decades due to their remarkable photosynthetic capabilities, high adsorption sites on cell surfaces, and internal bioaccumulation by various metabolites.

Cyanobacteria, a Gram-negative bacteria have a remarkable property for extracting metals from soil and water. These photoautotrophic species need light, carbon dioxide, nitrogen, phosphates, and vital metal ions for growth. Furthermore, they are responsible for a significant portion of global CO_2_ fixation. These species dominate freshwater, brackish, and marine environments as primary producers. Restricting nutrients such as phosphates control their growth and survival under these conditions (Wall et al., 2014; Singh et al., 2022; Singh et al., 2023). According to Gonzalez-Medina and Medina-Franco, (2019), the existence of active metabolites in cyanobacteria is known for its anti-inflammatory effects, free scavenging, photochemical quenching of reactive oxygen species (ROS), antioxidant activities, and suppression of cellular lipid autooxidation. Additionally, it uses biogenic metal processing to lessen the toxicity of heavy metal levels in aquatic habitats. De Bruijn (2016), also presented cyanobacteria as a bioremediation agent to remove hazardous waste from contaminated areas, including soil, water, wastewater, and sediments. Due to their strong affinity for soluble metals, they are crucial to the sequestration of metals (De Bruijn, 2016).

Through their interactions with these metal ions, studies have demonstrated that cyanobacteria significantly regulate the bioavailability of important heavy metals in water (Shen et al., 2021; Fawzy and Mohamed, 2017). They employ various coping mechanisms to deal with hunger and starvation of nutrients as they scavenge metals and store nutrients during the period of abundance. The remarkable characteristics of cyanobacterial species make them highly effective for utilizing biologically isolated trace metals in aquatic environments. These species can endure heavy metal-contaminated environments employing internal and/or extrinsic detoxification mechanisms (Al-Amin et al., 2021; Iqbal et al., 2022).

Cyanobacterial members are among the best prospects for the adsorption of heavy metals due to their distinct cell wall composition, comparatively large surface area, high mucin mass with high binding affinity, and low substrate requirements (Li et al., 2001; Rai and Tripathi, 2007). Furthermore, very little secondary waste is generated in most cyanobacterial bioremediation processes when hazardous heavy metal ions are removed (Kumar et al., 2021; Hooda et al., 2023).

Cyanobacteria are one type of microbe that is frequently employed in bioremediation and has a significant impact on the food and pharmaceutical industries. It is the least expensive way to get nutrients, including protein, carbohydrates, and necessary components. Despite the pharmaceutical applications, environmental bioremediation, and health benefits of cyanobacterial species, some are linked to undesirable tastes in drinking water. The release of geosmin and 2-methylisoborneol metabolites in the aquatic system could be responsible for this. According to Graham et al. (2010) and Merel et al. (2013), specific cyanobacterial blooms are also dependent on environmental conditions that emit cyanotoxins, which are known to be neurotoxins for the nervous system and the primary cause of skin irritation in humans.

This study investigates the effectiveness of metal bioremediation techniques that use photosynthetic cyanobacteria. Microbial cell metabolism and environmental use of biosorbents are closely related to the adsorption of metal ions in biological processes. Therefore, the ability of cyanobacteria strains to incorporate complex heavy metals into their inherent binding sites during the production of metabolites was carefully addressed in this investigation. Although the data is not readily available, we have made concerted attempts to link physiological reactions to ROS at the cellular level in their native habitat.

### Toxicity of heavy metal

When heavy metals such as Cu, Zn, Fe, Mn, Cr, and Co are present in nano-concentrations, they are regarded as micronutrients. However, they start to harm living things in higher quantities. Due to their intrinsic toxicity, a propensity to accumulate and biomagnification in food chains, and capacity to cause a wide range of pathophysiological conditions, including chronic diseases, such as dermatitis, stomach pain, and lung cancer in organisms, including humans, elevated levels of heavy metals have become a global cause for concern in the present scenario (Briffa et al., 2020; Karimian et al., 2021; Mitra et al., 2022). Metals are capable of causing diseases even at low concentrations.

Important trace metals, including Fe, Ni, Cu, and Zn, are essential components of enzymes and cytochromes, controlling metabolic processes. Ni, for instance, is a part of the urease enzyme and can be harmful to humans in excess (Mazzei et al., 2020). The metalloids As, Cd, Pb, and Hg are not essential to human survival or the normal functioning of other living organisms. Exposure to certain metals, even at low concentrations, causes a detrimental impact on human health. Using extensively contaminated water for irrigation may be the cause of the high levels of heavy metals found in food and the food chain. Approximately 90% of heavy metals are ingested via food and water, with the remaining 10% being contaminated through direct or indirect contact with a polluted environment or respiration. Many ailments can arise from the high concentration of essential and non-essential metals at trace levels because they interfere with enzyme function and disrupt metabolic processes in the body. While high quantities of zinc can cause cardiovascular and immunological diseases, trace amounts of zinc in the body regulate critical metabolic processes, including the content of high-density lipoprotein and various immunological functions (Karn and Pan, 2017).

According to Osae et al. (2023), exceeding the permissible amount of Nickel can harm gastrointestinal cells, impair red blood cell function, and reduce lung cell function. Similarly, elevated Pb levels may contribute to the development of neurological, cardiovascular, and mental disorders. According to the U.S. Environmental Protection Agency (EPA) regulation, the concentration of Pb in soil and water should be 50 and 15 μg/L, respectively. The higher concentration of Pb, more than the recommended limit, could affect the conformational structure of protein and nucleic acid, inhibition of oxidative phosphorylation, and enzyme activity (Karn et al., 2023). As per EPA guidelines, the concentration of Cr should be less than 2 mg/L in the environment. Cr in the environment exists in two oxidation states in the forms of Cr(III) and Cr(VI); Cr(VI) is found in the environment in a more mobile state whereas Cr(III) is less mobile, but both are toxic for a living being (Karn et al., 2018).

Due to the way that high amounts of Cd interact with bone, exposure can cause anomalies in the human skeleton. Furthermore, it is a contributing factor to the development of cancer. Carcinogenic As metal is the leading cause of cancer, dermal disorders, and respiratory complications. Hg also harms liver function and stomach illness (Rai et al., 2019). Table 1 details the source of metal contaminants, their impact on living organisms, and the metal removal efficiency of cyanobacteria.

**Table 1:** Sources of heavy metal and their bioremediation capacity of cyanobacterial species.

Metal	Source	Human health risk	Cyanobacterial species	Efficiency (%)	Reference
As	Dyes, pesticides, automobile, glass making industries	Skin degeneration, gastrointestinal diseases, vomiting	*Cyanobium* sp.	90	Pérez-Portilla et al. (2021)
Cd	Oil refineries, plastic industries, electroplating	Chronic kidney and liver disease, immune system damage	*Nostoc muscorum*	93	El Hameed et al. (2021)
Cr	Leather process industries, iron industries, paper and textile industries	Carciogenic, dysfunction of the nervous, reproduction, and circulatory systems	*Phormidium* sp. and *Oscillatoria* sp.	96	Shukla et al. (2012)
Cu	Mining industries, electrical appliances, industrial dust, fungicides	Anemia, hemolysis, jaundice, Wilson’s disease	*N. muscorum*	71	Goswami et al. (2015)
Hg	Oil refineries, byproducts of caustic soda, and chlorine production	Immunological disorder, damage to the brain	*Nostoc paludosum*	96	Franco et al. (2018)
Pb	Lead alloy, solder, plastic stabilizer	Degenerate skin and bones, acute kidney and liver diseases	*Gloeocapsa* sp.	65	Raungsomboon et al. (2008)
			*Nostoc commune*	97	Lavado-Meza et al. (2023)
Zn	Brass industries, Cosmetic, deodorant and electroplating	irritations of the skin, nausea and vomiting, and stomach upset	*N. muscorum*	66	Goswami et al. (2015)

According to Fein et al. (2019), Yu et al. (2020), and Chugh et al. (2022), the primary mechanism of metal toxicity in living organisms is the binding of hazardous metal to an active functional group of protein, such as sulfhydryl (SH^−^), carboxyl (COO^−^), or imidazole. This inhibits several physiological processes. This may increase the pace at which reactive oxygen species (ROS), the primary cause of oxidative stress, are formed. Due to electron leakage from the respiratory electron transport chain system, ROS may have developed faster when heavy metal concentrations increased and worsened under metal stress conditions (Nowicka, 2022; Drzeżdżon et al., 2018).

The accumulation of heavy metals results in reactive oxygen species (ROS), which harm cell membranes, decrease cellular respiration efficiency and impede the growth of microorganisms. The primary source of ROS formation is the generation of superoxide (O_2_^**−.**^), hydrogen peroxide (HOO**˙**), and hydroxyl (OH**˙**) radicals by them as well. In plants, chlorophyll is affected severely due to free radical-induced hypoactivation of enzymes, membranes, lipids, and nucleic acid (Jusoh et al., 2020; Ackova, 2018).

### Comparative bioremediation potential of cyanobacteria with other microorganisms, recovery, and reuse of the accumulated heavy metals

The aim of heavy metal bioremediation from contamination sites is to decrease the concentration of toxic metals or to reduce their toxicity by changing to oxidation number. Under the contamination of toxic metals, microorganisms combat their toxicity through biosorption and bioreduction processes by extracellular and intracellular defense mechanisms (Goswami et al., 2017). Most of the outer membrane of Gram-negative bacteria is composed of peptidoglycan, anionic lipopolysaccharide, and phospholipid, and these biomolecules have been found as active sites for heavy metal binding. For example, exopolysaccharides of *Pseudomonas aeruginosa* can chelate with heavy metals, and it increases with the concentration of toxic metal Cr(VI). Thus, the main strategies for the production of the high rate of extracellular polymeric substances (EPSs) are to prevent the entry of toxic metals into cells and release reductase enzyme that detoxifies the metal toxicity, Most of the microbial cell surface directly adsorb toxic Cr(VI) or it converts to Cr(III). The secretion of metabolites from microorganisms has immobilization and precipitation of heavy metals and increases the extraction efficiency from the contamination site (Guo et al., 2021).

Microbes in metal contaminants environments can precipitation in the form of insoluble metal, for example, biomineralization of Cd or magnetite from iron (Goswami et al., 2017). The mechanism of reducing the toxicity of metal is carried out by releasing precipitate agents such as phosphorus, carbonate, and sulfide. The microbes are able to reduce the solubility of Cd and form insoluble with this ligand, which acts as an anion donor (Zheng et al., 2021).

Scientific literature studies have shown that cyanobacteria have a greater ability than other biological microorganisms, such as fungi, yeast, and bacteria, to abate hazardous metals from contaminated sites. Due to certain cyanobacteria, such as *Phormidium, Anabaena*, and *Oscillatoria*, have distinct reactive functional groups on their cells that actively bond with heavy metals, they have a significant potential to remediate harmful metals from wastewater when it comes to microorganisms. They can, therefore, naturally reduce toxicity and thrive in water contaminated with heavy metals (Balaji et al., 2016). Table 2 provides an overview of the various microorganisms’ bioremediation capabilities.

**Table 2:** Bioremediation potential of some microorganism species.

Metal	Microorganism	pH	Efficiency	Reference
Cd	*Cupriavidus necator* (bacteria)	6	78.53%	Li et al. (2023)
	*Phlebia brevispora* (fungi)	6–7	77.3%	Sharma et al. (2020)
	*Chlorella vulgaris* (microalgae)	6	87.52%	Sayadi et al. (2019)
	*Spirulina platensis* (cyanobacteria)	6	92.76%	
Pb	*Oceanobacillus profundus* (bacteria)	6	97%	Mwandira et al. (2020)
	*P. brevispora* (fungi)	6–7	97.5%	Sharma et al. (2020)
	*C. vulgaris* (micro-algae)	6	90.09%	Sayadi et al. (2019)
	*S. platensis* (cyanobacteria)	6	94.09%	
Hg	*Bacillus cereus* (bacteria)	8	99 (mg/g)	Rani et al. (2021)
	*Lentinula edodes* (fungi)	6	337 (mg/g)	
	*C. vulgaris* (microalgae)	7	94.7 (mg/g)	
	*Aphanothece flocculosa (cyanobacteria)*	6	456 (mg/g)	Kalita and Baruah (2023)
Cr	*Pseudomonas aeruginosa* (bacteria)	6	0.625 (mg/g)	Kumar et al. (2021)
	*Rhizopus arrhizus* (fungi)	2	114.9 (mg/g)	
	*C. vulgaris* (micro-algae)	1.5	163.93 (mg/g)	
	*Spirulina plantensis* (cyanobacteria)	1.5	212 (mg/g)	Kalita and Baruah (2023)

Cyanobacteria are inexpensive to cultivate and maintain; they can even flourish in unfavorable environments. Growing cyanobacteria for dual purposes, such as removing hazardous metals and generating useful byproducts, including protein, carbohydrates, lipids, biogas, pigments, and biopolymers, is appropriate when using integrated approaches. According to Pekkoh et al. (2023), cyanobacterial species use CO_2_ as a nutrient for growth and enhance the rate of biomass production. As a result, this green gas decreases globally, and more valuable natural compounds are produced and used as raw materials in the pharmaceutical and cosmetics industries. Furthermore, cyanobacteria biomass may be stored, renewed, and used again for many years. Oxygenic photosynthesis is a unique property of cyanobacterial species, as evidenced by the increased oxygen concentration of their wastewater. According to Wu et al. (2017), it requires minimal light intensity, the fixation of nitrogen and carbon dioxide from the atmosphere, growth in metal-stressed conditions, a short growth period, and a high conversion rate of waste nutrients into valuable biomass. These unique characteristics provide these microorganisms an advantage over others in the bioremediation and heavy metal management of wastewater. More attention has been paid recently to the way that cyanobacteria absorb organic contaminants and their adaptable metabolic processes, which can be applied to bioremediation.

Furthermore, cyanobacteria are less expensive to cultivate than fungi and bacteria. In addition, because some cyanobacterial species employ carbon dioxide, nitrogen, and organic and inorganic phosphate as nutrients, they can be beneficial in treating industrial effluents.

Practically and technically, regenerating the biomass must be made before it is used for industrial purposes. Protons from the biomass in a low pH range exchange the binding sites to absorb heavy metal cations in the solution of the metal-loaded biomass. Ion exchange, which occurs at binding sites with protons and metal ions, is the main mechanism of metal ion desorption. Regarding the regeneration of the cyanobacterial biomass, cyanobacteria can be extracted from the contaminated aquatic site using straightforward filtering following the desorption process. Conventional filters can be utilized for filtration because of the large diameter. Additionally, additional cyanobacteria can be harvested for later use from the gathered material (Amano et al., 2024).

### The industrial aspect of cyanobacterium

A cyanobacterium has drawn greater attention among microbiological wastewater treatment techniques because of its high removal effectiveness, environmentally favorable metal pollutant degradation process, and ability to stabilize the ecosystem. Cyanobacterial species are often an inexpensive and lucrative operation method in industrial effluent treatment plants. For instance, cyanobacterial species from industrial secondary effluents, such as *Synechocystis* sp., and other microalgae species have been used to detoxify heavy metals. These species have been found to have a strong affinity for micropollutants such as the metals zinc and copper. It was found that these species in untreated form had 81.7% Cu and 94.1% Zn metal uptake capacity, whereas, the removal efficiencies enhanced up to 10% in the treated with autoclaved or dry biomass in single or combined form of these microbial strains (Chan et al., 2014). *Spirulina platensis* species biomass, when dried, immobilized, or live, may effectively remove harmful metals such as Cr^3+^ from tannery effluents (Shashirekha et al., 2008). *Oscillatoria* sp. has also demonstrated an impressive capacity for removing Cr^6+^ from contaminated aqueous solutions (Miranda et al., 2012).

Cyanobacterial species can serve as detoxification agents in tannery effluents and other contaminated industrial water bodies. For example, *Anabena flos-aquae* have a high degradation capacity of pollutant agents such as nitrogen. Similarly, the bioremediation and biodegradation of pollutants using *Nostoc* sp. and *Oscillatoria* sp. are highly effective in wastewater. These cyanobacterial species are dominant and able to grow in contaminated water (Dubay et al., 2011; Kannan et al., 2011).

### Bioremediation potential of the consortium of microorganisms

During wastewater purification, bacteria, fungi, microalgae, and cyanobacteria engage in symbiotic relationships (Solimeno and García, 2019). These microorganisms metabolize organic pollutants into carbon dioxide. Cyanobacteria use carbon dioxide generated in this process for photosynthesis, leading to an increase in biomass for metal accumulation. In addition, Cyanobacteria, microalgae, and bacterial consortium enhance the negatively charged functional group, such as carboxyl, amino, hydroxyl, and sulfide on the cell surface. Externally, these negatively charged groups are more affinity toward the toxic and heavy metals. Similarly, the intake of excess metals through aragonite (CaCO_3_) is a main constituent component of the structure of this consortium. Overconsumption of CaCO_3_, a significant component of the consortium’s structure, can lead to an intake of excessive metals. The consortium of *Chlorella sorokiniana* and *Ralstonia basilensis* possesses more copper-removal efficiency than an individual of this microorganism at pH 5, whereas other heavy metals, such as cadmium, nickel, and zinc, are less efficient as compared to copper (Muñoz and Guieysse, 2006). Dried biomass of a mixture of cyanobacteria (*Pseudoanbaena* sp., *Chroococcus* sp., and *Leptolyngbya* sp.) microalgae (*Tetraedron* sp., *Scenedesmus* sp., *Chlorococcus* sp., and *Chlorella* sp.) and diatoms (*Nitzschia* sp., *Navicula* sp., and *Cyclotella* sp.) are very efficient for removal of cadmium up to 100% (Loutseti et al., 2009).

### Live, dried, immobilization biomass-assisted bioremediation

The literature survey reported that the immobilized, live, and dead biomass of cyanobacterial species is effective for bioremediation. It is noted that both living and immobilized cells are more capable of metal remediation than dead cells. This may be due to the involvement of different remediation mechanisms. Metal ions are typically trapped on the surface of dead cyanobacterial cells. The existence of active binding sites and distinct cellular architecture could be the cause. Furthermore, the metabolic process has little effect on dead cells.

The incapability of metal ions to cross cell membranes in the absence of cellular metabolic cycles consequently reduces the efficacy of metal ion elimination. Similarly, metal ions uptakes by immobilized cells entrapped on the cell wall without any cellular metabolic process but increase the surface area of the cell. As a result, a higher bioremediation efficiency is exhibited than dead cells. Whereas, in the presence of a living cell, the metal ions entrapped on the cell surface and participate in the cellular metabolic process. Consequently, live cells have higher bioremediation effectiveness than dead cells. It could be stated that live as well as immobilized microalgae take up higher quantities of metal rather as compared to dead biomass.

According to some research, as compared to living stain, the maximal uptake of immobilized biomass of *Synechococcus* sp. on silica was found to be 143 mg/L Cu(II), 1,456 mg/L Pb(II), 142 mg/L Ni(II), and 529 mg/L Cd(II). Similarly, immobilizing a mixture of these cyanobacterial species, such as *Anabaena* var*iabilis* and *Tolypthrix ceytonica*, accelerates the uptake of Fe and Zn. In a comparable manner, it was discovered that *S. platensis* immobilized in calcium alginate had a greater divalent heavy metal removal efficacy than live biomass. Immobilization, which increases surface area and introduces new –OH and N–containing active functional groups to bind heavy metals, improves the bioremediation process (Bestawy, 2019; Gardea-Torresdey et al., 1998; Purev et al., 2023).

### Nature of water bodies on bioremediation process of heavy metal

In Indian hot springs, the cyanobacterial species such as *Scytonema ambikapurensis* have inorganic nutrient removal capacity for phosphate at 85%, sulfate at 65%, nitrate at 97%, ammonia at 95%, chloride at 70%, zinc at 80%, and at nickel 80%. As a result, utilizing this cyanobacterium not only reduces the levels of heavy metals but also improves the quality of the water by lowering ammonia levels, hardness of water, and increasing the quantity of biomass, carbohydrates, and chlorophyll (Jaiswal et al., 2023). It also produces vitamin B_12_ and exhibits a high binding ability with Mn. The biomass of cyanobacteria in this aquatic system increases at ambient iron levels. Moreover, *Dolichospermum lemmermannii* exhibits larger surface and volume ratios than diatoms due to the size of its phycosphere. Due to its increased cell volume and surface area, *D. lemmermannii* bioaccumulates metals in a higher proportion (Dengg et al., 2022). The substantial amount of nutrients in the artificial Kaunas Lagoon increases the bioaccumulation capacity of Cd by twenty-fold and Pb by approximately 2 times. Cyanobacterial species such as *Aphanizomenon flos-aquae* and *S. platensis* increase their bioaccumulation capacity of Cd by 20 times while Pb levels increase by approximately 2 times. Pb(II) and Cd(II) metal absorption capability relies on metabolic processes, protein carriers, and metal ion transport on cell membranes across cytoplasm. Similarly, As(V) is transported from the membrane to the cytoplasm by phosphate transporters, while As(II) is transported by glycerol transporters. Similarly, the transporters MerC and MerT proteins can transport Hg (Galinytė et al., 2023).

According to Winayu et al. (2024), thermophilic cyanobacterium *Thermosynechococcus* sp. CL-1 has a high CO_2_ fixation rate as a result of the rapid growth of biomass. In addition, this species generated valuable dominant byproducts such as C-phycocyanin pigment. This pigment has achieved high removal efficiency of Zn including Cd and Pb.

### Effect of CO_2_ concentration on metal uptake

High-value supplemental byproducts, including glycolipids, phycocyanins (PC), phycoerythrins (PE), carotenoids, fatty acids, and photoprotective substances such as mycosporine-like proteins can be obtained from cyanobacteria (Singh et al., 2017). They are also found to mitigate one of the important greenhouse gasses, that is, CO_2_, via photosynthesis-induced carbon fixation. The cyanobacterial biomass effectively increases due to enhanced oxygenic photosynthetic processes in the chloroplasts. The amounts of chromospheres, such as PC and PE are increased at higher concentrations of CO_2_. The most efficient removal of heavy metal by *Tolypothrix* sp. in the presence of CO_2_ is Al and V followed by Se > Cu > Zn > As > Ni > Sr. > Mo, while no significant effect of CO_2_ concentration for the removal of Mo, Fe, and Ni has been observed. *Oedogonium* sp. is the most effective species for the bioremediation of Al, Cu, Cr, Zn, and Ni in the presence of CO_2_. It may be due to an increase in the yield of *Oedogonium* sp. biomass in the presence of higher content of CO_2_ in wastewater (Roberts et al., 2015).

### Effect of pH, nutrient availability, temperature, light intensity, and competing metal ions on the bioremediation process of heavy metal

The pH is one of the most significant factors for the removal of metal ions from aqueous solution by adsorption. Generally speaking, pH is a crucial factor to take into account, particularly when screening for heavy metals employing immobilized cells. The sorbent made from cyanobacteria will be positively charged and have a stronger affinity for anions. However, above these values, the biosorbent will be negatively charged and have a preference for cations.

The cyanobacteria-based sorbent will be positively charged and have a stronger attraction for anions at acidic pH. However, the biosorbent will be negatively charged at these levels and will, therefore, prefer cations (Barquilha et al., 2019). It affects the rivalry between metallic ions, the chemistry of toxicants, and the activation of functional groups in cells (Das et al., 2008). This has an impact on the availability of the binding sites on the cyanobacterial cells’ surface, which has an impact on the cytoplasm’s osmotic potential and the entire photosynthetic electron transport system. The adsorbent surface charge, oxidation–reduction reaction, complexation reaction, and degree of ionization are all significantly influenced by the pH of the solution.

For instance, the pH level affects the existence of negative charges on the surrounding *Arthrospira platensis* biomass cells, which in turn affects the biosorption process of Hg(II). Among these, mercury Hg^2+^ is present in aqueous solution at pH 2, while Hg (OH)_2_ at pH 8. These crucial findings suggest that the dissociation of carboxylic and phosphoric groups are key factors for the determination of the acidic and basic behavior of biomass (Solisio et al., 2020). These acidic sites are –COO^−^, –NH_2_, and PO_4_^3−^, so they have different affinities for the uptake of Cr(VI) ions by *Nostoc muscorum* at pH 3. At low pH, negative charge density occurs in the biosorbent due to carboxyl and amino groups, and this negative charge intensively interacts with the Cr(VI) ion (Gupta and Rastogi, 2008).

The growth parameters such as nutrient and light intensity are likely to influence the constituents of the cyanobacterial cellular wall. So, the composition of the cells of the same strain cultured at different growth conditions acquires different biosorption capacities. For example, *Spirulina* sp. cell growth under photoautotrophic sequester Cr^3+^ is much higher than mixotrophic and heterotrophic conditions (Chojnacka et al., 2005). Prior to making the photosynthetic activity process under optimum light intensity, the cyanobacteria-mediated metal sequesters process increases due to the production of huge biomasses. These biomasses possess various valuable bioactive substances, which could be used for the metal bioremediation process.

The study of the impact of temperature on biosorption efficiency shows that the optimum temperature is important, and it varies from strain to strain. For example, in *Synechocystis* sp. it was found that the optimum temperature ranged from 20°C to 30°C and further it was decreased when the temperature was higher than 30° (Ozturk et al., 2014). Similarly, in *N. muscorum*, biosorption of Pb and Cd increases at temperatures from 10°C to 40°C, and above 40°C, it is drastically decreased. So, the biosorption efficiency of metal depends on the optimum temperature, and beyond this limit, it is going to decline. At the range of optimum temperature, the pores of cyanobacteria are enlarged with increasing temperature resulting in an increase in available surface area for the adsorption, penetration, and diffusion of metal within the pores. These results suggested that the biosorption of microorganisms with metals could be a chemical or physical adsorption. This may also occur due to either reducing the active sites of strain or destroying the biomass active sites (Sulaymon et al., 2013).

When comparing the metal removal efficiency in multiple metal ion systems by using biomass, there have been different affinities toward metal. It may be due to the individual metals having different redox potential, ionic radius, and electronegativity. For example, in *Oscillatoria* sp., the adsorption efficiency is the highest of the most electronegative metal as compared to low electronegative, and it may be due to these ions attracting strongly into the surface of biomass. Further, the biosorption efficiency for each metal is decreased; it may be due to competition among metals with active sites of biomass.

### Molecular insight into the heavy metal bioremediation process

At the industrial scale, enzymatic oxidation/reduction processes in *Cynobacteria* species are reported to be straightforward, quick, affordable, and effective for bioabsorption and bioaccumulation (Chojnacka et al., 2005). Mechanisms involved include trans-membrane transport, complexation on the cell surface, ion exchange, and physical adsorption. The metabolically independent binding of metals to cell walls and external surfaces presents in the case of an increase in living and non-living biomass. The bioabsorption capacity of the biosorbent may be affected by different parameters such as pH of the medium, incubation time, and acceleration by the presence quantity of nutrients in inorganic forms (Chojnacka, 2010).

Cynobacteria use a variety of ways such as cell walls express metal-binding proteins on their surfaces including internal metal-binding motifs ATPase that can absorb heavy metals such as Pb^2+^, Cd^2+^, and Zn^2+^ which are imported by transporters such as P-type Cd^2+^ ATPases and P-type Zn^2+^ ATPase in a strain of *Nostoc* sp (Ahad et al., 2021). It is possible for heavy metal ions to be captured and subsequently bioabsorbed into binding sites found in cellular structures on the cell surface of *Synechocystis* sp. The cyanobacteria presence of various anions in a constituent of *Synechocystis* sp., such as carboxylic, sulfonic, hydroxyl, and amine groups, interact with heavy metals such as Pb^2+^, Cd^2+^, and Cr^6+^. The various metabolites and components of cyanobacterial cells involved in metal uptake have been listed in Table 3.

**Table 3:** Cyanobacterial species and cellular components that are involved in the biosorption of heavy metals.

Cyanobacterial species	Component	Functional group/metabolite	Strategies/mechanism	Reference
*Nostoc muscorum*	Intracellular	Phytochelatin and activated PC synthase	PC synthases and vacuoles temporary storage	Greeshma et al. (2022)
*Calothrix* sp.	Exopolymer sheath	α-Carboxyl	Bonding with carboxyl sites of sheath and cell wall	Yee et al. (2004)
	Cell wall	*β*-Carboxyl		
	Reactive species between sheath and cell wall	phosphate, carboxyl, and amine ligands	Cu(II), Cd(II), and Pb(II) coordinate with ligand	
*Arthrospira platensis*	Thylakaloid membrane and pigments	Phycocinin, Allophycocinin	Chelating with Ni, Fe, and Zn	Cepoi et al. (2022)
*Anabaena* sp. strain Pasteur Culture Collection of Cyanobacteria (PCC), *Prochlorococcus marinus*, *Synechococcus* WH and *Thermosynechococcus elongatus* BP	ziaR/smtB- gene	Metallothionein ZiaA-like zinc exporter	The sequestration Cu, Co, and Zn	Cavet et al. (2003)
*Gloeothece magna*	Exopolymer sheath/cell wall	Extracellular polysaccharides	chelating with Cd(II) and Mn(II)	Mohamed (2001)
*Anabaena spiroides*	Exopolymer sheath/cell wall	Extracellular polysaccharides	Bind with Mn(II), Cu(II), Pb(II) and Hg(II)	Freire-Nordi et al. (2005)
*Anabaena flos-aquae*	Cell wall	Peptidoglycan	coordinated with amine and carboxyl ligands	Kretschmer et al. (2004)
*Anacytis nidulans*	Cytoplasmic cysteine-rich protein	Metallothionein	Cd(II) coordinate with Cystine	Yadav et al. (2020)
*Synechococcus* sp.				
*A. platensis*	Granules in vacuoles	Intracellular Polyphosphate	PO_4_^−3^accumalte Cd(II) and Zn(II)	Sanz-Luque et al. (2020)
*Microcystis aeruginosa*				
*Phormidium* (PA6)	Cell wall and cytoplasm	Cell wall and metabolite	Order of metal uptake as Ni > Cd > Pb > Cu with a range of78–98%	Naaz et al. (2021)
*Aphanothece* sp	Cell wall and cytoplasm	Cell wall of amino acids and organic acids	At lower temperatures, the maximum removal efficiency	Kalita and Baruah (2023)

At a higher concentration of Cd (30 mg/L), a clear detachment effect was observed between the mucilage external layer and cell membrane, which may be attributed to cell “plasmolysis” due to the toxic effects of Cd.

The recent bioremediation studies report shows that metallothionein and phytochelatins, a constituent compound of cyanobacterial, have a high affinity to metal binding and play a key role in metal homeostasis. Sun et al. (2024) demonstrated that the metallothioneins and phytochelatin-encoded genes in *Synechocystis* sp. strain PCC exhibit a high tolerance to multiple heavy metal ions and enhance the removed efficiency of Cd, Cu, and Zn from contaminated water. This encoded cyanobacterial species encapsulated with sodium alginate-based hydrogels also effectively removes the toxic Cd from animals, such as zebrafish and mice (Sun et al., 2024).

### Mechanism of metal uptake

Considering the ability to remove waste to an extensive degree, aquatic autotrophs have been utilized in both home and industrial settings to remove heavy metals and aid in the visualization of the detoxification process. It has been observed that the initial metal ions in aquatic autotrophs are absorbed on the surface and subsequently transported through the cytoplasm and cell membrane. The rate at which metal ions adsorb and absorption depends on several intrinsic and extrinsic parameters, including cell size, binding sites, cell organelles, temperature, pH, and competing ions in the surrounding environment (Deng et al., 2007). Cyanobacterial cells’ defense mechanisms, both enzymatic and non-enzymatic, alleviate the effects of mental stress. They used biosorption on the cell surface, intracellular bioaccumulation, and short-term compartmentalization to combat metal toxicity. Subsequently, they used biotransformation by various chelators, including phosphate anion, organic acids of proteins, hydroxy of polysaccharides, and finally, efflux through metal exclusion (Greeshma et al., 2022).

**Figure 1:**
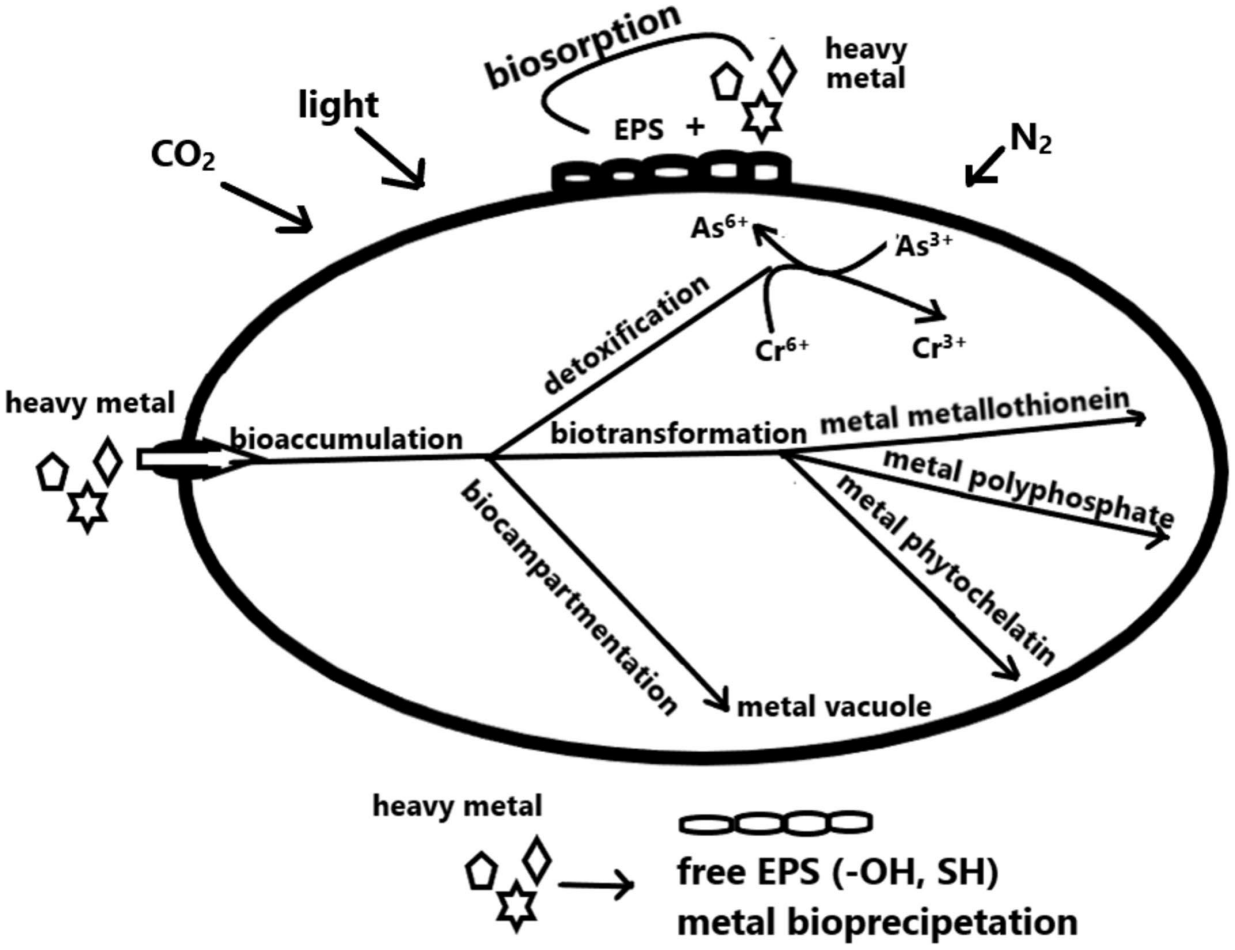
Mechanism of metal uptake by cyanobacterial cell.

The surfaces of cyanobacteria display intricate structures with various surface layers, each of which has a unique set of molecular functional groups and offers the most active sites for metal binding. This process deprotonates the ionic forms of heavy metals attached as metal–ligand surface complex compounds (Yee et al., 2004; Chojnacka et al., 2005). These proton-active functional groups include carboxyl (–COOH), amino (–NH_2_), phosphate (–PO_3_), hydroxyl (–OH), and sulfhydryl (–SH), which impart an overall net negative charge to the cell surface and result in a high binding affinity for metal catalysis with certain charged groups (Danouche et al., 2021). Of all the functional groups, carboxyl groups account for most of the active sites accessible to metal ions. Furthermore, one of the essential components for metal absorption in the protein compounds of cyanobacterial cell walls is the presence of amino groups (Kumar et al., 2015; Carreira et al., 2023). Figure 1 illustrates how complexation, chelation, ion exchange, and electrostatic interaction mediate the adsorption and accumulation of heavy metals in cyanobacterial cells.

Metals are accumulated by cyanobacteria through two mechanisms that occur both within and outside the cell. The initial uptake is quick, passive, and metabolically independent; it is mediated through the cell walls, in contrast to the cytoplasmic uptake process, which is active and metabolically reliant but comparatively sluggish. For instance, the diverse components found in the cytoplasm and cell walls of Anabaena species, including *Anabaena spiroides* and *Anabaena cylindrica*, allow their living cells to accumulate copper (Tien et al., 2005).

Metal ions swiftly adsorb onto the cell surface through electrostatic interaction whenever in contact with the heavy metal, a molecule accumulates during the passive and active absorption. After being absorbed through the cell membrane through an active absorption process reliant on metabolism, ions are subsequently dispersed throughout the cytoplasm. A plasma membrane-mediated transport of metal ions into cellular compartments necessitates the presence of living cells for intracellular enrichment and is frequently linked to a cell’s defense mechanism against a toxic metal (Fischer et al., 2019). The “detoxification” of the mobile metal species can then proceed through enzymatically redox-driven reactions, precipitation of metal ions by cell components (biomacromolecules), oxidation state transformation in the cytoplasm, or a combination of these processes.

Predominantly cyanobacteria are known to produce metal-binding proteins in the cytoplasm in response to the presence of metal ions which sequester them in biologically inactive, non-cellular toxic forms, such as cysteine-rich metallothioneins (Li et al., 2021). For example, the comparative studies between living and non-living *Spirulina* sp. for adsorption efficiency of Cd(II) show that the living species are a better option than non-living due to higher enzymatic activities in live species (Doshi et al., 2007). In the *Microcystis aeruginosa* (Tien et al., 2005) non-living cell extracts do adhere to the same passive adsorption mechanisms as living cell wall adsorption. The passive adsorption mechanism is occasionally reversible and can involve external functional groups from both living and non-living cases (Kumar et al., 2015).

### Alteration of metabolite synthesis during the metal uptake process

Essential components of cyanobacteria are enzymes, metals, and metalloids that regulate various catalytic actions and maintain protein structure, which affects the biological systems. Cyanobacteria’s high levels of metals and metalloids deal with homeostasis and tolerance mechanisms to reduce the dysfunctional effects. Furthermore, elevated concentrations of heavy metals in cyanobacteria compensate by changing the activity of antioxidant enzymes such as glutathione (GSH) peroxidase, catalase (CAT), ascorbate peroxidase, and superoxide dismutase (SOD). Additionally, it also affects the synthesis of photosynthetic pigments such as chlorophyll and carotenoids. In particular, Anabaena species with elevated amounts of nickel and arsenic can maintain by lowering their malondialdehyde (MDA) concentration and boosting the activity of antioxidant enzymes within their cells. These physiological mechanisms suggest that they are an adaptive defense mechanism adopted under metal stress conditions. Similarly, CO_2_ fixation activity is slowed down due to altering the ribulose-1,5-biphosphate carboxylase-oxygenase (RuBisCO) large subunit (rbcL) enzyme in the Calvin cycle under dark reactions, which shows that adoptive mechanism under nickel stress condition in *Anabaena* sp. The inhibitory mechanism of CO_2_ fixation is further demonstrated by the change in the activity of the fructose 1,6-bisphosphatase enzyme (Prajapati et al., 2020). High concentrations of Cd metals have been shown to enhance *γ*-glutamylcysteinyl glycine (GSH) levels in cyanobacteria, revealing that Cd metals chelate with phytochelatin to reduce metal toxicity. Under Cu(II) stress conditions, *Synechocystis* sp. forms Cu-EPS and Cd-EPS complexes at high Cd concentrations and increases extracellular protein concentrations, indicating resistance to metal toxicity (Shen et al., 2021). Microorganisms such as yeast, bacteria, and fungi are usually utilized to sequester metal from soil and water bodies. However, cyanobacteria are a particularly promising bioagent because of their high photosynthetic activity, low nutrient requirements can survive in adverse conditions, ease of cultivation, large surface area, and large capacity to produce EPS, which have a greater affinity for metal cations and reduce the amount of metal that enters the cell. *Cyanothece* sp. responds to metal stress by producing more thylakoid vesicles and altering the distance between intra-thylakoid membranes. Additionally, heavy metals alter the redox reaction and modify the electron transport chain, which is influenced by the structure of the thylakoids (Mota et al., 2015).

### Specific strategies for enhancing uptake of heavy metal

The ability of cyanobacterial species to sequester harmful metals through byproducts without compromising their primary biological functions gives them an edge over other species. When hazardous elements such as Pb and Cd are present in wastewater, *C. sorokiniana* increases its respiration rate because of reduced photosynthetic activity. It appeared to demonstrate that *C. sorokiniana*’s high respiration rate maintains cellular metabolism. Higher copper ion concentration significantly lowers the amount of phycobilisomes in *Synechocystis aquatilis*. According to Dudkowiak et al. (2011), there is no discernible effect of Cd, Zn, and Pb cations on the content of chlorophyll and phycobilisomes in *S. aquatilis*, but there is a significant influence of Hg, Ag, and Cu toxicity. Certain cyanobacterial species have been shown to generate cyanotoxins, a biocomponent of microcystins with a strong affinity for metal ions, and may form complexes with Cu, Mo, and Mn, but not Zn. The synthesis of microcystin in cyanobacteria is dependent on the cellular need for metal for appropriate physiological function. Therefore, the synthesis of toxins is affected by increasing metal concentrations (Larsona et al., 2023). Certain cyanobacterial species are best suited to accumulate heavy metals in high concentrations of heavy metals when given specific nutrient supplement conditions. For example, *Nostoc* sp. has a higher affinity for Ni than for Cu and Zn and a high metal affinity when maltose is supplied. These cyanobacteria may efficiently accumulate more metal from wastewater because they produce significant quantities of EPSs by adding maltose (Ghorbani et al., 2022). P nutrient levels in aquatic systems promote the formation of cyanobacterial blooms when they are present in abundance. Phosphorus is a fundamental structural element of cyanobacteria and regulates a wide range of functional aspects of metabolic activities, including the formation of adenosine triphosphate (ATP), DNA, RNA, and P storage vacuoles. The cyanobacterial cells absorb an ambient amount of phosphorus through their outer membrane, which is then transported to the plasma membrane, across the periplasm by binding proteins, and finally to the cytoplasm. The synthesis of polyphosphate (polyP) in cyanobacterial species stores internal phosphate, which is then used by the enzymes exo-polyphosphatase and polyP kinase. Polyphosphate’s role as a metal chelator may be responsible for intracellular phosphate storage (Xiao et al., 2022).

### Oxidative stress, enzymatic alteration of heavy metal bioremediation

It is well established that the cyanobacterial species manage or scavenge the reactive oxygen species (ROS) with the help of secondary metabolites and antioxidant enzymes. These non-enzymatic and enzymatic antioxidants are counterattack against the ROS (Boden et al., 2021). For example, antioxidants enzymes such as glutathione reductase (GR), SOD, and CAT. In the cyanobacterial species, various SODs enzymatic cofactors such as CuSOD for copper, ZnSOD for zinc, FeSOD for iron, NiSOD for nickel, and MnSOD for manganese are used for scavenging ROS. Non-enzymatic antioxidants such as glutathione, proline, thiol, and phytochelatin concentration increase in cyanobacteria species such as *Nostoc entophytum* and *Plectonema boryanum* when it is exposed to high concentrations of Cd^2+^ (Ahad et al., 2021). The antioxidant enzyme maintains the redox pool in microbial cells and establishes a dynamic equilibrium between the utilization and synthesis of GSH.

It is the most abundant non-protein thiol in cyanobacterial species. As a result, the GSH content rises in response to high concentrations of toxic metal exposure to combat oxidative stress. It was discovered that the cyanobacterial cell’s increased GSH synthesis was caused by the GSH precursor cysteine. As a result, cyanobacterial cells have enormous amounts of GSH, making them tolerant to heavy metals’ toxicity. Despite having high intracellular metal concentrations, these species did not suffer serious consequences from metal stress. Additionally, GSH polymerizes to form phytochelatin that chelate with toxic metals by its thiol (SH) groups (Jozefczak et al., 2012).

The non-enzymatic antioxidant metabolites, such as proline, thiol, and phytochelatin concentration, significantly increase under Hg stress conditions (Saini et al., 2021). These biocompounds are also referred to as metallophytes or hyperaccumulators due to their extraordinary capacity to absorb heavy metals when exposed to high concentrations of toxic metals or other unfavorable conditions such as high temperatures, droughts, and salinity, which raise proline concentrations in cyanobacterial microorganisms (Singh et al., 2015). Proline shields several vital enzymes from stress because of its unique physical and chemical structure. It also aids in stabilizing cell membrane systems, participates in chlorophyll synthesis, and has an adaptive mechanism for heavy metal tolerance (Zhou et al., 2020).

The capacity of *N. muscorum*, *S. platensis*, *Synechococcus* strain PCC 7942, and *Synechocystis* cyanobacterial species to tolerate heavy metal stress and eliminate ROS toxins occurs as a result of an upsurge in both non-enzymatic proline and enzymatic (GSH, GR, ascorbate peroxidase, CAT, SOD). As a result, cyanobacterial species are able to endure stress, proliferate, defend against heavy metal-induced ion leakage of cell membranes, and shield cells from heavy metal oxidation (Tripathi and Sahu, 2023). As(V) and As(III) in oxyanions are two forms of arsenic, which are hazardous metals that can be obtained from artificial and natural sources. It is one of the most toxic metals and is detoxified by the nicotinamide adenine dinucleotide phosphate (NADPH)-dependent flavoprotein system, a redox enzyme, which transfers an electron to a protein substrate or an additional electron acceptor, such as quinones or dithiols. Thus, these enzymes play a crucial role in biosynthesis, biochemical decomposition, and metal detoxification reactions. Arsenical resistance mechanisms are performed by ArsH proteins which are encoded by the genes arsB, arsC, and arsH in *Synechocystis* sp. (Hervás et al., 2012).

### Exopolysaccharide and heavy metal bioremediation

EPSs are generated in accordance with the conditions of the environment, stress, and strain. Generally, EPSs are produced by cyanobacterial cells to defend themselves against biotic and abiotic stresses. In recent years, it has been recognized as high-value molecules with a unique composition and flexible structure. Currently, researchers are using EPS for a wide range of applications, including in the food and pharmaceutical sectors and bioremediation which is helpful in removing heavy metals from contaminated water. The extracellular polymer of cyanobacteria is mostly composed of heteropolysaccharides that contain substituents other than carbohydrates, such as uronic acids, fatty acids, and peptides (Parwani et al., 2021; Tiwari et al., 2020).

In recent years, EPSs have drawn major interest from researchers due to their metal binding affinity, water solubility, and ease of recovery from liquid cultures (Mota et al., 2016). For example, even in the presence of other heavy metals, ESPs from *A. spiroides* have been shown to exhibit a high and specific chelating capacity with Mn^2+^ heavy metals (Gupta and Diwan, 2017).

**Figure 2:**
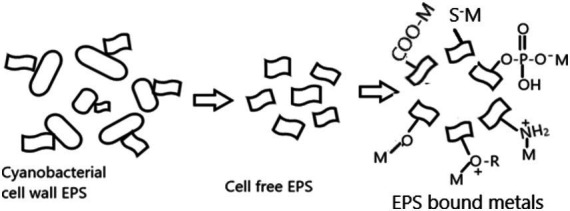
Mechanism of metal ion biosorption process in EPS.

EPS biomolecules contain diverse ionizable functional groups, such as sulfate, phosphoric, carboxylic, amino, and hydroxylic groups, which show a high affinity for heavy metallic elements, and these groups bind with metal ion through electrostatic, coordination, complexation, or precipitation (Mouro et al., 2024). The constituent of EPS can be found in less condensed forms or densely condensed forms. Less condensed elements are loosely attached to the cells and partially released and dissolve in the surrounding medium. Dense condensed constituents create a thick coating or capsule that envelops the cell. Figure 2 shows the mechanism of various heavy metals biosorption of EPS.

The biosorption mechanism involves the fast, reversible, and passive binding of EPS with heavy metals and is now available for loosely connected EPS (Ciani et al., 2024; Cui et al., 2021). The polyelectrolyte negative charges component on EPS has been considered a chelating agent for positively charged heavy metals from contaminated sites. Due to the high surface-to-volume ratio of EPS in cyanobacterial species, the dense capsule also produces galacturonic acid, which has a high affinity toward chelate cations for binding to Cd(II), Cu(II), Fe(III), Ni(II), and Zn(II).

The capacity of biosorption of metal through EPS also depends upon the availability of the constituent’s specific group, and it differs from strain to strain. For example, *Gloeothece* sp. amide and carboxyl groups are found to have the highest binding capacity with Cu in comparison with other metals (Micheletti et al., 2008). Likewise, *Anabaena doliolum* preferred to biosorption of Cd among other metals in contaminated water due to the presence of hydroxyl, carboxyl, amides, carbonyl, and sulfate groups (Al-Amin et al., 2021).

It is found that specific functional groups of EPS are active at particular pH conditions for the biosorption of metal. For example, the carboxyl group active at pH 2–5, the carboxyl and phosphate group involved at pH 5–9, carboxyl, phosphate, amine, and hydroxyl group metal sequestration at pH 9 from contaminated water (Amano et al., 2024). ESPs from cyanobacteria were reported to display maximum affinity for heavy metals among other microorganisms tested so far. For example, *Anabaena oryzae* species released polysaccharides that have an affinity to Zn exclusion and are ascribed as Zn accumulation (Chakraborty and Mishra, 2020). Uronic acids, which are sugars containing sulfate, have a strong anionic character and a high affinity for metal cations (Ohki et al., 2014). The polysaccharide content of uronic acid, galacturonic acid, and glucuronic acid in *Synechocystis* sp. BASO671 is markedly elevated because of the presence of Cd(II), Cr(VI), and a solution of Cr(VI) and Cd(II). The ability of cyanobacteria-derived ESPs to detoxify heavy metals from wastewater has been amply demonstrated (Ozturk et al., 2014).

Extracellular polysaccharides in *Synechocystis* sp. are made up of humic acid, while extracellular proteins are rich in tryptophan and tyrosine amino acids. When Cr(VI), Pb(II), and Cd(II) are present, *Synechocystis* sp. exhibits an enormous rise in polysaccharide contents along with a modest increase in extracellular protein. Thus, to accumulate metal ions, ESPs are favored over proteins (Cui et al., 2021).

### Metallothioneins and metal uptake

The function of MTs and phytochelatin is to sequester heavy metals in vacuoles. Heavy and toxic metals are transported through organic ligands across the cell wall to the cytoplasm and compartmentalized in vacuoles (Balzano et al., 2020). MTs are the low molecular weight cystine rich peptides, which are found in cyanobacterial species such as *Synococcus* sp (Lavorante et al., 2003). They are thermally stable metal-sequestering proteins that have the ability to attach to highly toxic metals such as Pb, Cd, Hg, and As, both essential and non-essential, and form metal-thiolate clusters. Most cyanobacterial species produce MTs, which adhere to heavy metals and assist in detoxifying their host cells while fostering homeostasis. Cyanobacteria have developed a variety of methods, such as the metal chelating proteins and MT as a phytochelatin, to preserve metal homeostasis while reducing the adverse consequences of excess metal ions (Chatterjee et al., 2020). The class-II MT-like proteins in cyanobacterial sp. encoded by the smtA in which heavy metals make easy absorption and rapid growth. The smtA is a zinc-induced gene present as a zinc-tolerance gene in *Synechococcus* sp. strain PCC 794. This gene has four zinc ions bound by the His-imidazole and Cys-sulfur ligands (Balzano et al., 2020).

### Polyphosphate and heavy metals uptake

In cyanobacterial species, polyphosphate, an orthophosphate polymer with a phosphorus-anhydride link, is believed to be a stress-resistant metabolite. Numerous physiological functions, including protein binding, phosphate storage, metal ion excretion, intracellular stress management, and biofilm formation, are facilitated by cellular polyphosphates (Sanz-Luque et al., 2020). The heavy metal phosphate compounds are compartmentalized in the vacuole and also activate the system for metal efflux. The metabolism and compartmentalization of polyphosphate are regulated by acidocalcisome membrane and exopolyphosphatase enzymes. Acidocalcisomes have a strong affinity for essential metals such as Mg^2+^, Ca^2+^, Zn^2+^, and Fe^3+^ owing to their acidic nature. Hence, polyphosphates and acidocalcisomes play a key role in the homeostasis process of essential metal cations (García-García et al., 2016).

The acceptability of metal tolerance in cyanobacteria is related to elevated polyphosphate concentration and suggests that it can sequester heavy metals by intracellular concentrations in cyanobacteria cells. More polyphosphates are also stored as polyphosphate granules, which are then hydrolyzed to produce phosphate, precipitating in metal forms and detoxifying the host cells (Chandwadkar and Acharya, 2023). The polyphosphate correlated with toxic metal concentration and their localization patterns in various forms such as granules of Cu(II) polyphosphate in extracellular matrix, Pb(II) polyphosphate crystals in periplasm and inclusions of Cr(III), Pb(II) and Zn(II) polyphosphate in cytoplasm, overall presenting a high metal sequestration capacity (Villagrasa et al., 2021).

When starved, carbon-containing cyanobacterial cells undergo internal polyphosphate metabolism, releasing phosphate precipitating as a metal phosphate on the cell wall. An increase in the number of polyphosphate bodies with heavy metal toxicity was also observed in cyanobacteria, as polyphosphate bodies act as an indicator of metal uptake capacity. The strong negative surface charge of polyphosphates in the phosphate body can help with metal accumulation. According to Sanz-Luque et al. (2020), the polyphosphate body may have two functions: it can be a storage polyphosphate and contribute to detoxification.

The exposure of *Chlamydomonas reinhardtii* to Hg^2+^ containing solution initiates the degradation of polyphosphate and, therefore, increases the amount of short-chain polyphosphate and orthophosphate in the vacuoles as the metal ions are being sequestered (Samadani and Dewe, 2018). It implies that longer polymers and/or the energy in phosphor anhydride bonds may contribute to greater sequestration effectiveness, corresponding to that observed in *Synechocystis* sp. When phosphate is present, an efficient biological oxidation in *Synechocystis* sp. changes highly toxic As(III) to less lethal As(V) (Zhang et al., 2013). Consequently, cyanobacterial cells absorb As(V) via phosphate transporters, while the glycerol protein system transports arsenic metal in a lower oxidation state (Rossen, 2002).

### Cyanobacterial pigments assisted bioremediation

The agricultural and medicinal industries have an enormous demand for cyanobacterial photosynthesis-derived pigments, including phycobiliproteins and carotenoids (Saini et al., 2018). Phytochelatin (PC) is a phycobiliprotein, a component of phycobilisomes and is bound with thylakoid chloroplasts. The pigment phycocyanin interacts strongly with metal ions among the phycobiliproteins. When the Cd and Pb concentrations spike, the PC content in *S. platens* increases significantly however, the As concentrations had no noticeable impact. Although the precise nature of the metal binding sites with phycocyanin is unknown, it is most presumably the globular structure, negative electrostatic binding with cation, and binding site with thiol group of cystine or hexamer of phycocyanin (Galinytė et al., 2023).

Thermodynamically, the heavy metal binds to cyanobacterial pigments due to negative free energy. Furthermore, the interaction with C-phycocyanin is endothermic for Cd^2+^, Cu^2+^, and Zn^2+^ along with positive entropy. Therefore, under endothermic enthalpy, compensating is not beneficial. In fact, the unfolding and expanding of PC occur due to interactions of entropically absorbed energy (Celus et al., 2018). The positive enthalpy has been found favorable for the binding of heavy metal ions Hg^2+^ (Ngu-Schwemlein et al., 2005) and Fe^3+^ (Grossoehme et al., 2006) to PC, suggesting a strong interaction between metal ions and pigments. The positive value of entropy may be due to the helical structure of PC, resulting in an increase in the total randomness of the same and more adsorption of heavy metal ions over it. Checchetto et al. (2013) reported that in *A. cylindrica*, the thylakoid membranes of PC absorb photons, resulting in variations in fluorescence. This could lead to further PC pigment synthesis and increased sequestration of Pb^2+^ metal ions over time.

### The current and the future challenges of cyanobacteria-based heavy metal sequestration

Drawing inspiration from scientific research, it has been discovered that some cyanobacterial species are a valuable source of products. Considering they are involved in metabolic pathways, metabolism, and proliferation, cyanobacterial species have an advantage over other conventional approaches in the wastewater treatment process. Therefore, additional research is required to extend the analytical approach related to the productivity of cyanobacterial biomass, and the future strategies ought to be developed at the molecular level. Furthermore, it is critical to identify specific bioaccumulation-inhibiting factors, such as a reliable biosorption process.

However, the potential of cyanobacterial byproducts has not been completely abundant in the marketplace to the present. Furthermore, the diversity and abundance might alter other microbial densities in the ecosystem and the wastewater’s chemical and physical properties. From a biological perspective, physiological aspects must be extensively studied and utilized. To convert desired metabolites efficiently, consideration should be given to cyanobacteria harvesting, their bioremediation performance, and comparisons with other microorganisms.

Thus, the effectiveness of bioremediation depends on specific ecological parameters, and tests of adaptability in particular environmental conditions are required for heavy metal uptake. The enormous diversity and resilience in extreme environmental conditions must be considered while screening and identifying promising cyanobacterial species for heavy metal absorption from wastewater. Moreover, there are still limitations and restrictions on collecting cyanobacterial blooms in commercials.

Anthropogenic activity accelerates the rate of nutrients in water bodies, which encourages the synthesis of secondary metabolites. These secondary metabolites may be cyanotoxins, which are harmful to both humans and aquatic life. Thus, certain cyanobacterial species generate cyanotoxins, which are toxic compounds, compared to other microorganisms. These cyanotoxins are neurotoxins, and hepatotoxins have detrimental effects on ecosystems and pose a global danger. These toxins can inhibit plant growth, accumulate in food, and inhibit other microorganisms from growing (Ramakrishnan et al., 2023). Analyzing every cyanobacterium attribute at the genomic level to produce cyanotoxin compounds is difficult.

From a technical standpoint, it is crucial to break down these cyanotoxins using Fenton-type processes, which employ transition metals such as Fe and Cu in the presence of carbon, ascorbic acid, H_2_O_2_, and UV light. These metals bind the cyanotoxin, which breaks down by their interaction with a macrocyclic ligand, in addition to numerous bonds, and electron transfer processes (Schneider and Bláha, 2020).

These methods are effective in degrading cyanotoxin, but some practical limitations exist due to slower degradation processes. Additional cyanobacterial bioremediation processes are slower than traditional treatment processes. Furthermore, the excessive use of toxic cyanobacterial blooms is a threat to ecosystems. For example, *Anabaena circinalis*, *Nodularia spumigena*, *M. aeruginosa*, *Cylindrospermopsis raciborskii*, and *Planktothrix* sp. produce cyanotoxins that are dangerous for other aquatic living organisms (Dittmann et al., 2013). From a technical standpoint, if it is necessary to use toxic species for the bioremediation process, it puts the dead cell under optimum temperature to remove toxicity for a safe alternative for the removal of metals.

Further, the quality of water may be disturbed due to the high density of cyanobacterial species and their secreted extracellular polymeric components, which have a high affinity toward the surface of the filter unit and interfere with the filtering process (Romanis et al., 2021). Therefore, the primary focus for growing forward should be managing cyanobacterial blooms peculiar to a certain site. In addition, there exist numerous technological obstacles concerning the environmental applications and benefits associated with cyanobacteria, including the identification and taxonomic assessment of their functional capacities in real cultures.

## Conclusion

In addition to generating large quantities of beneficial metabolites, cyanobacterial cells can withstand the toxic effects of heavy metals in contaminated growth media. The efficiency of biosorption and bioaccumulation, as well as the sequestration of heavy metals on the surface and inside of cells, were also evaluated in this study. Besides biological components, abiotic variables, such as determining the optimal pH, temperature, and photonic conditions in appropriate combinations, can also significantly impact the design of effective bioremediation processes.

We have gone over how the adsorption interactions between metal ions and metabolites are influenced by the pH of the solution as well as the metabolites’ properties. An anticipated enhancement in adsorption efficiency holds significant potential for impactful pollution management in the realm of bioremediation.

Cyanobacterial metabolites effectively mitigate the toxic effects of metals in aquatic environments; further development and emphasis on alternative pathways in cyanobacterial metabolomics are imperative to enhance the efficiency of bioremediation.
